# Application of Acupoints and Meridians for the Treatment of Primary Dysmenorrhea: A Data Mining-Based Literature Study

**DOI:** 10.1155/2015/752194

**Published:** 2015-02-24

**Authors:** Siyi Yu, Jie Yang, Mingxiao Yang, Yan Gao, Jiao Chen, Yulan Ren, Leixiao Zhang, Liang Chen, Fanrong Liang, Youping Hu

**Affiliations:** ^1^Chengdu University of Traditional Chinese Medicine, No. 37 Shierqiao Road, Jinniu District, Chengdu, Sichuan 610075, China; ^2^Chengdu University of Information Technology, Chengdu, Sichuan 610225, China; ^3^Second Traditional Chinese Medicine Hospital of Sichuan Province, Chengdu, Sichuan 610031, China

## Abstract

*Background.* Dysmenorrhea is a common problem for which acupuncture provides effective analgesia. Although acupoint selection affects the effectiveness of acupuncture, the basic rules of acupoint selection are little understood. This study aims to investigate the principles of acupoint selection and characteristics of acupoints used for primary dysmenorrhea. *Methods.* PubMed, China National Knowledge Infrastructure, and Chinese Biomedical Database were searched for clinical trials published in English or Chinese from January 1978 to April 2014 evaluating the effect of acupuncture on primary dysmenorrhea, with or without methods of randomization and/or control. Three authors extracted information and two reviewers inputted information on titles, journals, interventions, main acupoints, and outcomes using the self-established Data Excavation Platform of Acupoint Specificity for data mining. *Results. Sanyinjiao* (SP06), *Guanyuan* (CV04), and *Qihai* (CV06) were used most frequently. The most frequently used meridians were Conception Vessel, Spleen Meridian of Foot *Taiyin*, and Bladder Meridian of Foot *Taiyang*. 67.24% of acupoints used were specific acupoints. Acupoints on lower limbs were most frequently used. *Conclusion.* Data mining is a feasible approach to identify the characteristics of acupoint selection. Our study indicated that modern acupuncture treatment for primary dysmenorrhea is based on selection of specific acupoints according to traditional acupuncture theory.

## Introduction

Dysmenorrhea is a common problem for adolescent girls and women of reproductive age [1]. Primary dysmenorrhea (PD) is medically defined as those menstrual periods when females feel pain without any organic change in the uterus. The typical symptom is a pain that originates in the lower abdomen and spreads to the inner thighs; this can cause agony for a couple of days every month [2]. The prevalence of dysmenorrhea appears different across the world, ranging from 80% in Western Australia [3] to 60% in Canada [4], 48.4% in Mexico [5], and 79.9% in Iran [6]. One estimate depicted that 51% of young women reported limited activities and another 17% had sick leave from either school or work because of severe menstrual pain [4]. Hence, the burden of dysmenorrhea is greater than any other gynecological complaint and has a significant impact [7].

Common treatments for dysmenorrhea include rest (58%), medications (52%), heating pads (26%), tea (20%), exercise (15%), and herbal medicine (only 14%) [8]. The most frequently applied therapy to treat PD is nonsteroidal anti-inflammatory drugs (NSAIDs) [9]. However, there are often unpleasant adverse events associated with NSAID use, including stomachache, diarrhea, and nausea [9]. This has led to increasing numbers of patients seeking complementary and alternative techniques such as acupressure and moxibustion to treat symptoms of PD [10].

Acupuncture is an important technique in the field of Traditional Chinese medicine (TCM), used for both disease prevention and therapy. However, evaluation of acupuncture in clinical trials that could accelerate TCM modernization has not been possible until the past few years. With the introduction of evidence-based medicine (EBM), domestic and foreign scholars have carried out a lot of scientific research into acupuncture. It is now well known that the randomized controlled trial (RCT) is the gold standard for efficacy and safety assessment. Through the comprehensive collation of relevant RCTs, one systematic review found that acupuncture was associated with a significant reduction in pain compared with pharmacological treatment or herbal medicine for PD [11]. Another systematic review indicated that acupuncture was superior to both no-acupuncture control and sham acupuncture for the treatment of chronic pain, suggesting that factors in addition to the specific effects of needling are important contributors to therapeutic effects [12].

The development of EBM has caused acupoint specificity to become the research focus. Acupoint specificity is of prime importance in point selection, and it involves specificity in terms of clinical effect, biological structure, and biophysics. According to the principles of TCM, the selection of acupoints plays a vital role in the therapeutic effectiveness of acupuncture.

The selection of acupoints is changeable according to the different ideas and experience of the acupuncturists. There is a vast volume of literature related to acupuncture and moxibustion; however, as the diversity between different schools is huge, determination of the most effective selection and application of treatment is very difficult. Thus precise information describing the optimal selection and combination of acupoints for the treatment of PD is not presently available.

Fortunately, the emergence of data mining techniques allows new ways of analyzing acupuncture and moxibustion information. Data mining, which is also referred to as knowledge discovery in databases, is the nontrivial extraction of implicit, previously unknown, and potentially useful information from data [13]. To date, our team has fundamentally researched the application of meridian points for the treatment of poststroke disorder [14], migraine [15], and functional dyspepsia (FD) [16] based on data mining technology. Proper use of data mining can acquire new knowledge hidden in vast amounts of TCM information, enabling the design of more rigorous RCTs to validate results. For example, one study analyzed the application characteristics and laws of acupoints in the treatment of FD and found that* Zusanli* (ST36) was the most frequently used acupoint [16]. Another study also found that acupuncture on* Zusanli* (ST36) of the Stomach Meridian group is effective in the treatment of FD and is superior to the Gallbladder Meridian group [17]. This trial may provide evidence for the existence of specificity between acupoints on different meridians and that the benefit of acupuncture relies on acupoint specificity. Therefore determining the characteristics and rules of acupoint selection for PD is important for future research and in clinical practice.

The aims of this study were to (1) discover the basic rules of acupoint selection in meridians and specify acupoints in different body parts used for treating PD in modern literature and (2) investigate the association rule of acupoints based on data mining, so as to provide relatively standard treatment guidelines in the application of meridian points for PD.

## 2. Methods 

### 2.1. Search Methods

PubMed (http://www.pubmed.com/), China National Knowledge Infrastructure (CNKI) (http://www.cnki.net/), and Chinese Biomedicine Database (CBM) (http://www.sinomed.ac.cn/) were searched for literature on acupuncture treatments for PD from January 1978 to April 2014.

The search strategy combined the key words (i) “acupuncture” or “electroacupuncture” or “moxibustion” or “meridian” or “acupoint”; and (ii) “primary dysmenorrhea” or “dysmenorrhea” or “menstrual pain.” The search included the literature on acupuncture treatment for secondary dysmenorrhea. Only electronic databases were searched for eligible studies. Language was restricted to English and Chinese.

### 2.2. Review Process

#### 2.2.1. Data Screening

*(1) Types of Studies.* Inclusion criteria included clinical trials evaluating the effect of acupuncture, with or without methods of randomization and/or control. The number of participants had to be more than ten in each group/trial. The final publication was used in the case of duplicate publications.

Exclusion criteria included reviews, animal trials, case reports, systematic reviews, and meta-analyses. 

*(2) Types of Participants.* Inclusion criteria included clinical trials involving participants diagnosed with PD.

Exclusion criteria included trials evaluating the therapeutic effect of acupuncture for secondary dysmenorrhea caused by endometriosis, uterine myoma, endometrial polyps, pelvic inflammatory disease, and other gynecological problems. 

*(3) Types of Intervention.* Inclusion criteria were as follows. The treatments for PD had to involve needle insertion and/or moxibustion at either traditional meridian acupoints or extraordinary acupoints. Electrical stimulation of needles was included. Acupuncture and/or moxibustion was either used alone or in addition to other interventions (e.g., Chinese herbs). Trials that compared different forms of acupuncture for PD were included.

Exclusion criteria included studies investigating modern methods of stimulating acupuncture points without needle insertion (e.g., laser stimulation or transcutaneous electrical stimulation). Trials of microacupuncture systems were also excluded, as the theoretical basis of microacupuncture has no relevance to traditional acupoints. Trials evaluating acupressure and trials stimulating pain points or trigger points alone for PD were excluded. 

*(4) Types of Outcome Measurements.* Inclusion criteria were as follows. Studies were included if they reported at least one clinical outcome related to dysmenorrhea (e.g., response, frequency, pain intensity, menstrual symptom scale, or analgesic use). In the case of controlled trials, studies included were those in which patients treated with acupuncture alone or in combination showed more benefits than patients who did not get acupuncture therapy. If a study compared the therapeutic effects of different acupoint prescriptions, the most effective acupoint prescription was included.

Exclusion criteria were as follows. Trials reporting only physiological or laboratory parameters were excluded. In the case of controlled trials, studies were excluded if patients treated with acupuncture alone or in combination showed fewer benefits than patients who did not get acupuncture therapy. If a study compared the therapeutic effects of different acupoint prescriptions, acupoint prescriptions other than the most effective one were excluded.

#### 2.2.2. Data Collection

All abstracts identified by the literature search were screened by Siyi Yu, who excluded those that were clearly irrelevant (e.g., studies focusing on reviews, animal trials, case reports, and so on). Full texts of all remaining references were obtained and again screened to exclude irrelevant papers. The eligibility of all other articles was then formally checked by Jie Yang and Mingxiao Yang according to the abovementioned selection criteria. Disagreements were resolved by discussion.

#### 2.2.3. Data Preprocessing

Information on titles, journals, interventions, main acupoints, and outcomes was inputted independently by Yan Gao and Jiao Chen using the self-established Data Excavation Platform of Acupoint Specificity (Copyright Registration number 2009SR014647) for data mining. As many acupoints have aliases, the names of acupoints were standardized according to* Fundamentals of Acupuncture*.

#### 2.2.4. Data Processing

**Figure 1:**
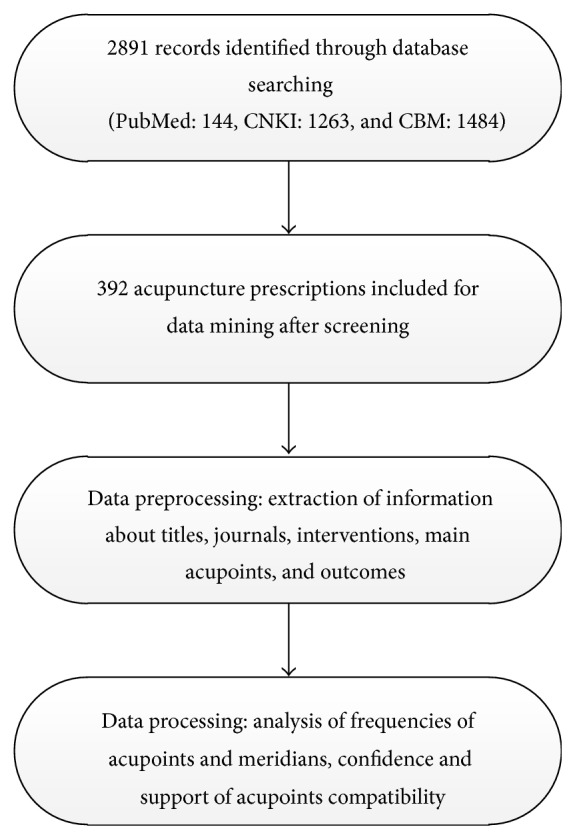
Flow of information through the different phases of data mining.

Based on the data mining algorithm of multihierarchy rules, related knowledge of selection and combination of acupoints can be acquired by calculating the frequency, support degree, confidence, and list level of acupoint item sets. Support degree is an index to describe the probability that events A and B synchronize under specific conditions; this was used to measure the statistical significance of association rules within the entire dataset. The coverage of an association rule is the number of instances for which it predicts correctly; this is often called its support. Its confidence or accuracy is the number of instances that it predicts correctly, expressed as a proportion of all instances to which it applies [18]. Support displays antecedent support, that is, the proportion of instances for which the antecedents are true, based on the training data. Confidence displays the ratio of rule support to antecedent support. Lift displays the ratio of confidence for the rule to the prior probability of having the consequent. In general, rules with lift different from 1 will be more interesting than rules with lift close to 1. Association rules are useful only when the support degree and confidence level meet the minimum requirements. See Figure 1 for a flow diagram of data processing steps taken.

## 3. Results

### 3.1. Overall Profile of Acupuncture Prescriptions

Database searching identified 144 records in PubMed, 1263 records in CNKI, and 1484 records in CBM. After filtering, a total of 392 acupuncture prescriptions were included in this study (see Figure 1).

### 3.2. Application of Acupoints

This analysis aimed to provide the acupoint selections and their frequencies when curing certain diseases. The most frequently used acupoints for PD in descending order were* Sanyinjiao* (SP06),* Guanyuan* (CV04),* Qihai* (CV06),* Diji* (SP08),* Ciliao* (BL32),* Zusanli* (ST36),* Taichong* (LR03),* Xuehai* (SP10), and* Shenshu* (BL23) (see Table 1).

### 3.3. Application of Meridians

Meridian application analysis demonstrated how the selected acupoints in the prescription were distributed in the 14 channels, including the frequency and percentage of the acupoints on each meridian, the number and percentage of acupoints used, and the name and frequency of each acupoint. The selected acupoints were distributed among 13 meridians, including 11 regular meridians, Governor Vessel, and Conception Vessel. The most frequently used meridian was Conception Vessel, with Spleen Meridian of Foot* Taiyin* and Bladder Meridian of Foot* Taiyang* also frequently used. Extraordinary acupoints were also frequently used. The frequencies for each meridian and acupoint are shown in Table 2.

### 3.4. Application of Special Acupoints

The results of the analysis depicted how certain acupoints are used in acupuncture prescriptions, including the frequency of the main categories, the numbers, and the type of acupoint used. Seventy-eight of the 116 acupoints used were specific acupoints, accounting for 67.24% of the total number of acupoints. The majority of the special acupoints used were Crossing acupoints, with meridian qi passing through and crossing in the abdomen; Front-*Mu* acupoints, Five* Shu* acupoints, Yuan-Source acupoints, and Back-*Shu* acupoints were also frequently used (see Table 3).

### 3.5. Application of Acupoints on Different Body Parts

The results of the analysis displayed the frequency and percentage of the distribution of acupoint selections in the prescriptions, the numbers and percentages of acupoints used, and the names and frequencies of particular acupoints. Acupoints on lower limbs were most frequently used, with 33 acupoints used a total of 839 times. This was followed by acupoints on the chest and abdomen (used 752 times), back and lumbar acupoints (used 409 times), acupoints on the upper limbs (used 93 times), and acupoints on the head, face, and neck (used five times) (see Table 4).

### 3.6. Association of Acupoint Compatibilities

The aim of the analysis was to indicate the compatibility of acupoints where the number of selected acupoints in the prescription was equal to or more than two, with the effectiveness of the compatibility measured by support degree and confidence level. The top acupoint pairing was* Guanyuan* (CV04) and* Sanyinjiao* (SP06). The support degree indicates that* Guanyuan* and* Sanyinjiao* appeared together in the 392 prescriptions 60.97% of the time, while the confidence level suggests that* Guanyuan* and* Sanyinjiao* appeared together in the related prescriptions 60.70% of the time. Lift was different from 1 in all results, indicating that the results predicted by the rules were reliable. The combination of the acupoints* Sanyinjiao* (SP06),* Guanyuan* (CV04),* Qihai* (CV06),* Ciliao* (BL32),* Zhongji* (CV03), and* Diji* (SP08) was used most frequently, as indicated by the support degree and confidence level meeting the minimum requirements. The 10 most frequently used acupoint compatibilities and their support, confidence, and lift are shown in Table 5.

## 4. Discussion

### 4.1. The Characteristics of Data Mining Applied to Acupuncture and Moxibustion

In data mining, the databases used for acupuncture and moxibustion have many unique attributes compared to those of other types. The purpose of data collection is to enhance patient treatment as well as making the information available source for research purposes. The difficulties associated with data mining for acupuncture and moxibustion treatments are as follows.

#### 4.1.1. Ambiguity

TCM therapy involves the physician choosing a treatment based on syndrome differentiation after acquiring pertinent information. The ambiguity of data refers to not only the treatment forms but also the fact that many symptoms are not individually named or one name is not solely related with a single symptom. Consequently, physicians may use different terms to describe the same symptom.

#### 4.1.2. Privacy

The data relating to acupuncture and moxibustion inevitably involves some private patient information. It is the obligation and responsibility of researchers mining data to conduct scientific research based on protecting the privacy of the patients and ensuring data security and confidentiality.

#### 4.1.3. Redundancy

There can be a lot of entirely or partially repeated information in the huge data source relating to acupuncture and moxibustion, some of which may be immaterial or contradictory.

#### 4.1.4. Complexity

In the field of acupuncture and moxibustion the data can be discrete, continuous, or hybrid, making noise processing rather complicated. The mining process needs human-computer interaction and multiple replications, with professional expertise required at every step.

To summarize, the data mining of acupuncture and moxibustion treatments is an interdisciplinary subject fraught with difficulty. There must be cooperation between acupuncture scholars and information technology professionals for success. The aim is to make some breakthroughs in coalescing related information in multidimensional attributes, increasing the efficiency and accuracy of mining algorithms.

### 4.2. Application Characteristics of Meridian Points for PD

The selection and combination of acupoints plays an important role in the effectiveness of PD treatments. The therapeutic effect of acupuncture is dependent on appropriate acupoint selection and combination.

#### 4.2.1. Great Importance Attached to* Yin* Acupoints and Meridians

The three most frequently used acupoints were* Sanyinjiao* (SP06),* Guanyuan* (CV04), and* Qihai* (CV06), all of which belong to* yin* meridians.* Sanyinjiao* (SP06) belongs to the collection of distal acupoints, which are located below the elbows and knees. As the junction point of three* yin* meridians of the foot,* Sanyinjiao* is heavily related to the Thoroughfare Vessel, Conception Vessel, and uterus. Needling* Sanyinjiao* can affect and promote the flow of qi and blood so as to improve the nourishment of the Thoroughfare Vessel, Conception Vessel, and the uterus and can relieve menstrual pain.* Sanyinjiao* is commonly used for gynecologic indications in clinical practice, especially for alleviating dysmenorrhea [19–21].* Guanyuan* (CV04) and* Qihai* (CV06) are local acupoints, which means that they are acupoints located in the affected area. The three* yin* meridians of the foot meet the Conception Vessel at* Guanyuan* and the Thoroughfare Vessel comes into confluence with the Kidney Meridian in the abdomen.

According to modern literature on the acupuncture treatment of PD, acupoints on the Conception Meridian were most frequently used. The Conception Meridian is a* yin* meridian; it is also one of the eight extra meridians, which act as reservoirs of Qi and blood for the 12 regular channels, filling and emptying as required, and provide further connections between the twelve regular channels. Furthermore, the Conception Vessel starts from the uterus and emerges from the perineum. The acupoints on this particular meridian have specific therapeutic effects on the body parts that the meridian runs along and connects with.

#### 4.2.2. Emphasis on the Specific Points

Specific acupoints recorded for the treatment of PD exceeded nonspecific acupoints in both number and frequency. Crossing acupoints, Front-*Mu* acupoints, Five* Shu* acupoints, Source-Yuan acupoints, and Back-*Shu* acupoints were the most frequently used acupoint types. The larger number and higher frequency of specific acupoints are in accordance with their specific therapeutic effects. The Crossing acupoints refer to those located at the intersection of two or more meridians. Since meridians converge at Crossing acupoints, the Crossing acupoints can be used to treat disorders of multiple meridians. Front-*Mu* acupoints are located on the chest and abdomen, while Back-*Shu* acupoints are located on the back. Each zang organ and fu organ are associated with one Front-*Mu* and one Back-*Shu* acupoint, respectively. These Front-*Mu* and Back-*Shu* acupoints are the areas where visceral qi infuses. Five* Shu* acupoints are used to treat diseases located on the regions over which the meridians run along the surface of the body. Additionally, source points are closely related to the zang-fu organs and are the points where visceral qi is infused via the Triple Energizer. Therefore, disorders of zang-fu organs can be treated by needling source points.

#### 4.2.3. Priority Given to Distal Acupoint Selection

Acupoints on the lower limbs were much more frequently used than acupoints on other parts of the body. The frequent use of distal acupoints is consistent with the principle of distal acupoint selection. Distal curative effect is a feature of the acupoints on the fourteen meridians. These distal acupoints can not only be needled to treat disorders of the regional tissues but also can be used to treat viscera, tissues, and organs associated with the meridians they are located on. Some of the distal meridian acupoints can even be needled to treat disorders of the whole body, thus explaining the saying that “the indication extends to where the meridian reaches.”

### 4.3. Acupoint Compatibility in Treatment of PD

Categories of acupoint combinations include local acupoint combinations, distal-proximal acupoint combinations, exterior-interior acupoint combinations, and anterior-posterior point combinations. These results from modern literature discovered via data mining might shed some light on the selection of acupoints and meridians for PD in clinical practice and scientific research.

However, needling compatible acupoint combinations can increase treatment effectiveness. The combination of such acupoints as* Sanyinjiao* (SP06),* Guanyuan* (CV04),* Qihai* (CV06),* Ciliao* (BL32),* Zhongji* (CV03), and* Diji* (SP08) was most frequently used in modern literature and might have better therapeutic effects than other less frequently used or unused acupoint combinations for PD. Compatibility of acupoints is considered to have a synergistic effect, which can enhance the therapeutic effect of acupuncture. For instance, one study showed that the therapeutic effect of needling* Shiqizhui* (EX-B8),* Sanyinjiao* (SP06),* Ciliao* (BL32), and* Diji* (SP08) in combination provided superior analgesia 10 min after needle insertion compared with needling* Shiqizhui* (EX-B8) alone [22].

Conversely, an antagonistic effect of acupoints might exist, as with TCM herbs. Although no evidence supporting acupuncture therapy for PD being effective has been found in related literature, an animal trial indicating that electroacupuncture can improve gastrointestinal movement in rats found that the effect of needling* Pishu* (BL20) alone was better than the effect of needling* Pishu* (BL20) and* Zusanli* (ST36) at the same time [23]. A lot of acupuncturists adopt the principle that the more acupoints needled the better curative effect can be achieved. However therapeutic effect is not always synchronized with a high number of acupoints needled, of which some may be unnecessary. Unfortunately, the use of a complicated acupuncture combination without self-limitation may hinder the synergistic effect and may cause an antagonistic effect that could worsen the clinical symptoms.

### 4.4. Limitations

Although the results of data mining can be useful to acquire new knowledge, there are still limitations.

First, in the modern acupuncture literature with randomized controlled evidence, those involved evidence-based medicine methodologies are lacking. Hence in this study the quality of the included studies was not evaluated. This may affect the scientific quality and objectivity of the results owing to the inconsistent quality of the literature.

Second, since the study information was not standardized, some studies failed to report the specific treatment time, treatment frequency, and amount of stimulation. Some descriptions of the outcome measurements were also unclear or confusing in the classification of syndromes.

## 5. Conclusions

In this study, data mining was applied to identify the most frequently used acupoints, meridians, special points, and the distribution of acupoints, as well as the correlating rules for selecting acupoints in practice for treating PD. The most frequently used acupoints were* Sanyinjiao* (SP06),* Guanyuan* (CV04), and* Qihai* (CV06). The most frequently used meridians were Conception Vessel, Spleen Meridian of Foot* Taiyin*, and Bladder Meridian of Foot* Taiyang*. The majority of acupoints used were specific acupoints (67.24%), with acupoints on the lower limbs being most frequently used. Our findings indicate that* Sanyinjiao* (SP06),* Guanyuan* (CV04),* Qihai* (CV06),* Ciliao* (BL32),* Zhongji* (CV03), and* Diji* (SP08) should be investigated further in forthcoming trials or used in clinical practice for PD.

Data mining is a feasible and applicable approach for researchers to identify the characteristics of acupoint selection. Our study indicated that selection of specific acupoints according to traditional acupuncture theory serves as the basis of modern acupuncture treatments for primary dysmenorrhea.

## Figures and Tables

**Table 1:** 20 most frequently used acupoints identified by data mining.

Number	Acupoint	Frequency	Support (%)
1	*Sanyinjiao* (SP06)	257	65.56
2	*Guanyuan* (CV04)	234	59.69
3	*Qihai* (CV06)	142	36.22
4	*Diji* (SP08)	126	32.14
5	*Ciliao* (BL32)	126	32.14
6	*Zusanli* (ST36)	123	31.38
7	*Zhongji* (CV03)	120	30.61
8	*Taichong* (LR03)	114	29.08
9	*Xuehai* (SP10)	77	19.64
10	*Shenshu* (BL23)	73	18.62
11	*Shenque* (CV08)	70	17.86
12	*Hegu* (LI04)	58	14.80
13	*Zigong* (EX-CA1)	49	12.50
14	*Guilai* (ST29)	43	10.97
15	*Mingmen* (GV04)	42	10.71
16	*Shuidao* (ST28)	40	10.20
17	*Ganshu* (BL18)	36	9.18
18	*Yinlingquan* (SP09)	32	8.16
19	*Shiqizhui* (EX-B7)	30	7.65
20	*Pishu* (BL20)	27	6.89

**Table 2:** Meridians and acupoints used in acupuncture therapy for PD.

Number	Meridian	Frequencies	PCT (%)	Acupoints		
				Number	PCT (%)	Selected acupoints and their frequencies
1	CV	608	27.92	11	10.47	*Guanyuan* (CV04) 234, *Qihai* (CV06) 142, *Zhongji* (CV03) 120, *Shenque* (CV08) 70, *Zhongwan* (CV12) 16, *Qugu* (CV02) 8, *Yinjiao* (CV07) 5, *Danzhong* (CV17) 4, *Shimen* (CV05) 3, *Xiawan* (CV10) 3, and *Shuifen* (CV09) 3

2	SP	501	23.01	9	8.57	*Sanyinjiao* (SP06) 257, *Diji* (SP08) 126, *Xuehai* (SP10) 77, *Yinlingquan* (SP09) 32, *Gongsun* (SP04) 4, *Daheng* (SP15) 2, *Fujie* (SP14) 1, *Yinbai* (SP01) 1, and *Taibai* (SP03) 1

3	BL	365	16.77	23	21.90	*Ciliao* (BL32) 126, *Shenshu* (BL23) 73, *Ganshu* (BL18) 36, *Pishu* (BL20) 27, *Geshu* (BL17) 19, *Shangliao* (BL31) 17, *Zhongliao* (BL33) 14, *Xialiao* (BL34) 12, *Weishu* (BL21) 7, *Chengshan* (BL57) 5, *Zhiyin* (BL67) 5, *Guanyuanshu* (BL26) 4, *Weizhong* (BL40) 3, *Qihaishu* (BL24) 3, *Jueyinshu* (BL14) 3, *Dachangshu* (BL25) 2, *Zhibian* (BL54) 2, *Heyang* (BL55) 2, *Kunlun* (BL60) 1, *Baihuanshu* (BL30) 1, *Feishu* (BL13) 1, *Zhishi* (BL52) 1, and *Sanjiaoshu* (BL22) 1

4	ST	243	11.16	11	10.47	*Zusanli* (ST36) 123, *Guilai* (ST29) 43, *Shuidao* (ST28) 40, *Tianshu* (ST25) 22, *Fenglong* (ST40) 7, *Wailing* (ST26) 1, *Shangjuxu* (ST37) 1, *Neiting* (ST44) 2, *Daju* (ST27) 1, *Dubi* (ST35) 1, and *Qichong* (ST30) 2

5	LR	139	6.38	8	7.62	*Taichong* (LR03) 114, *Xingjian* (LR02) 8, *Ququan* (LR08) 6, *Zhangmen* (LRl3) 3, *Qimen* (LR14) 3, *Ligou* (LR05) 2, *Zhongdu* (LR06) 2, and *Zhongfeng* (LR04) 1

6	EX-HN	79	3.63	2	1.90	*Zigong* (EX-CA1) 49, *Shiqizhui* (EX-B7) 30

7	GV	63	2.89	10	9.52	*Mingmen* (GV04) 42, *Yaoyangguan* (GV03) 10, *Dazhui* (GV14) 3, *Baihui* (GV20) 2, *Zhongshu* (GV07) 1, *Jinsuo* (GV08) 1, *Yaoshu* (GV02) 1, *Xuanshu* (GV05) 1, *Jizhong* (GV06) 1, and *Shuigou* (GV26) 1

8	LI	63	2.89	3	2.86	*Hegu* (LI04) 58, *Quchi* (LI11) 4, and *Shangyang* (LI01) 1

9	KI	60	2.75	12	11.42	*Taixi* (KI03) 24, *Yindu* (KI19) 17, *Zhaohai* (KI06) 8, *Dahe* (KI12) 3, *Yongquan* (KI01) 1, *Youmen* (KI21) 1, *Huangshu* (KI16) 1, *Siman* (KI14) 1, *Yingu* (KI10) 1, *Shuiquan* (KI05) 1, *Fuliu* (KI07) 1, and *Qixue* (KI13) 1

10	GB	26	1.19	8	7.62	*Yanglingquan* (GB34) 16, *Xuanzhong* (GB39) 4, *Xiaxi* (GB43) 1, *Fengchi* (GB20) 1, *Tinghui* (GB02) 1, *Daimai* (GB26) 1, *Zulinqi* (GB41) 1, and *Jingmen* (GB25) 1

11	PC	19	0.87	2	1.90	*Neiguan* (PC6) 18, *Zhongchong* (PC9) 1

12	LU	5	0.23	2	1.90	*Lieque* (LU07) 4, *Kongzui* (LU06) 1

13	HT	4	0.18	2	1.90	*Shenmen* (HT07) 3, *Shaofu* (HT08) 1

14	TE	2	0.09	2	1.90	*Zhongzhu* (TE03) 1, *Waiguan* (TE05) 1

*Note.* CV stands for Conception Vessel, SP stands for Spleen Meridian of Foot *Taiyin*, BL stands for Bladder Meridian of foot *Taiyang*, ST stands for Stomach Meridian of Foot Yangming, LR stands for Liver Meridian of Foot *Jueyin*, EX-HN stands for extraordinary point, GV stands for Governor Meridian, LI stands for Large Intestine Meridian of Hand Yangming, KI stands for Kidney Meridian of Foot Shaoyin, GB stands for Gallbladder Meridian of Foot Shaoyang, PC stands for Pericardium Meridian of Hand *Jueyin*, LU stands for Lung Meridian of Hand *Taiyin*, HT stands for Heart Meridian of Hand Shaoyin, and TE stands for Triple Energizer of Hand Shaoyang. Frequencies of meridians mean that the total frequency is of acupoints on the same meridian. PCT means the percentage of a specific meridian frequency accounting for the total frequency of all meridians. The number of acupoints means the total number of selected acupoints on the same meridian. PCT of acupoints means the percentage of number of acupoints accounting for the total number of selected acupoints in all meridians.

**Table 3:** Frequencies and numbers of different types of acupoints.

Number	Special point	Frequencies	Number
1	Crossing point	783	29
2	Front-*mu* point	406	9
3	Five-*mu* point	353	25
4	Yuan-source point	200	5
5	Back-*shu* point	150	8
6	Lower *he*-sea point	143	4
7	*Xi*-cleft point	130	4
8	Eight confluent points	62	6
9	*Luo*-connecting point	36	6
10	Eight convergent points	36	6

**Table 4:** The frequencies and numbers of acupoints on different body parts.

Number	Body part	Frequencies	Number
1	Lower limbs	839	33
2	Chest and abdomen	782	30
3	Back and lumbar	439	27
4	Upper limbs	93	11
5	Head, face, and neck	5	4

**Table 5:** Statistics of the 10 most frequently used acupoint combinations.

Number	Combination of acupoints	Support (%)	Confidence (%)	Lift
1	*Guanyuan* (CV04) →*Sanyinjiao* (SP06)	60.97	60.70	1.12
2	*Sanyinjiao* (SP06) →*Guanyuan* (CV04)	54.08	68.40	1.12
3	*Sanyinjiao* (SP06) →*Qihai* (CV06)	28.06	81.82	1.34
4	*Guanyuan* (CV04) →*Qihai* (CV06)	28.06	80.00	1.48
5	*Sanyinjiao* (SP06) →*Ciliao* (BL32)	27.04	71.70	1.18
6	*Guanyuan* (CV04) →*Zhongji* (CV03)	26.53	65.39	1.21
7	*Sanyinjiao* (SP06) →*Zhongji* (CV03)	26.53	65.39	1.07
8	*Sanyinjiao* (SP06) →*Diji* (SP08)	25.77	82.18	1.35
9	*Guanyuan* (CV04) →*Diji* (SP08)	25.77	59.41	1.10
10	*Guanyuan* (CV04) →*Qihai* (CV06), *Sanyinjiao* (SP06)	22.96	76.67	1.42
